# Impact of health system interventions to improve access to external beam radiotherapy: a scoping review

**DOI:** 10.3389/fonc.2026.1786405

**Published:** 2026-06-17

**Authors:** David Chen, Karren Xiao, Russell Leong, Kristen Arnold, Angela Doyle, Fabio Ynoe de Moraes, Paris-Ann Ingledew, Srinivas Raman

**Affiliations:** 1 Princess Margaret Hospital Cancer Center, Radiation Medicine Program, Toronto, ON, Canada; 2 Temerty Faculty of Medicine, University of Toronto, Toronto, ON, Canada; 3 Department of Radiation Oncology, BC Cancer Vancouver, Vancouver, BC, Canada; 4 Faculty of Medicine, University of Ottawa, Ottawa, ON, Canada; 5 BC Cancer Library, Vancouver, BC, Canada; 6 Division of Radiation Oncology, Department of Oncology, Kingston General Hospital, Queen’s University, Kingston, ON, Canada

**Keywords:** accessibility, EBRT, equity, external beam radiation therapy (EBRT), health system intervention, sustainability

## Abstract

**Purpose:**

Access to external beam radiotherapy (EBRT) remains heterogeneous across global health systems, particularly in low- and middle-income countries (LMIC). This scoping review aimed to synthesize evidence on health-system interventions intended to improve EBRT access, characterize their outcomes, and identify promising strategies for sustainable and equitable radiotherapy delivery.

**Methods:**

A systematic search of Ovid MEDLINE, Embase, and CINAHL from inception to June 30, 2025, identified studies evaluating system-level interventions to improve EBRT access. Eligible interventions included infrastructure expansion, workforce development, operational redesign, telemedicine, financing, and quality improvement initiatives. Data were extracted in duplicate and narratively synthesized by intervention domain.

**Results:**

Across 31 included studies, the most frequent intervention domains were infrastructure expansion (45%), workforce development (35%), operational redesign (29%), telemedicine (26%), referral and navigation (19%), and financial reforms (10%). The majority of studies reported improved radiotherapy utilization, reduced waiting times, and higher treatment completion rates. Multi-component programs that integrate interventions such as infrastructure and workforce expansion, operational redesign, tele-radiotherapy, decentralization through hub-and-spoke models, and protocolization of hypofractionation regimens, workforce, and operational redesign, can be a sustainable strategy to progress towards equitable, system-level programs to improve EBRT access.

**Conclusion:**

Health-system interventions can meaningfully expand access to EBRT when they align investments in infrastructure, workforce, and operations. Multi-component, equity-focused strategies hold promise for scalable, high-quality radiotherapy delivery to address access disparities. Future research should adopt standardized access metrics and report equity-stratified outcomes to guide national strategies for sustainable scale-up of EBRT.

## Introduction

Radiation therapy is an essential component of modern cancer care, given that approximately half of all cancer patients will require external-beam radiotherapy (EBRT) during the course of their disease (1). In high-income countries, radiotherapy is integrated into standard oncology guidelines with high rates of utilization. In contrast, access to EBRT remains inequitable across health systems globally, with particularly severe capacity and delivery gaps in many low- and middle-income countries (LMICs), rural regions, and otherwise underserved populations (2). For example, in certain low-income settings, it has been shown that fewer than one-quarter of eligible patients receive EBRT, leading to preventable morbidity and excess mortality (3).

The drivers of this gap in access to EBRT are multifactorial. A shortage of infrastructure persists, with many countries lacking radiotherapy machines and related resources. Even where facilities exist, equipment downtime and limited maintenance capabilities can undermine the reliability of radiotherapy service delivery. Workforce shortages further constrain access in rural areas, due to insufficient training of medical professionals in these regions as well as concentration of expertise in urban centers, often leaving rural patients underserved. Financial barriers also substantially reduce accessibility in healthcare systems without publicly funded support or compassionate access. These barriers collectively reinforce inequities in which cancer patients receive timely treatment and the overall quality of their cancer care.

Recognizing this crisis, academic institutions, governments, and international organizations including the World Health Organization (WHO) and the International Atomic Energy Agency (IAEA) have advanced guidance and trialed a range of system-level interventions to improve access to EBRT. In particular, WHO–IAEA guidance has emphasized that sustainable radiotherapy scale-up depends not only on procurement of equipment, but also on infrastructure expansion, workforce training and task-shifting, tele-radiotherapy programs enabling remote contouring and peer review, financing reforms to improve affordability, and service delivery innovations aimed at increasing machine throughput and reducing waiting times (4, 5). Collectively, initiatives designed to scale EBRT in LMICs could save millions of life-years and generate net economic gains (1, 6).

Despite these calls to action, the evidence base for health-system interventions remains fragmented. Many published studies of quality improvement in radiation oncology are single-center, short-term, and heterogeneous in design (7). To address this gap, centralized databases to assess radiotherapy capacity, such as the International Atomic Energy Agency (IAEA) Directory of Radiotherapy Centers (DIRAC), are useful for benchmarking radiotherapy infrastructure, equipment, and service distribution worldwide (8). There remains the need to design standardized outcomes based on radiotherapy utilization, equity, or waiting times, stratified based on socioeconomic status, geography, or gender barriers to access in order to systematically map and optimize radiotherapy access (9). Consequently, policymakers often rely on pilot or institutional data with non-standardized outcomes when designing national radiotherapy programs.

To address this knowledge gap, we conducted a scoping review to synthesize evidence on health-system interventions aimed at improving access to EBRT. By collating published evaluations across infrastructure, workforce, financing, and service delivery domains, our goal was to identify strategies with promising effectiveness, highlight contextual factors influencing success, and provide evidence to guide policymakers on relevant factors that may impact access to EBRT.

## Methods

We conducted this scoping review guided by the JBI Manual for Evidence Synthesis, reported in accordance with the PRISMA 2020 and PRISMA-ScR guidelines (Appendix 1), and prospectively registered with PROSPERO (CRD420251058737) (10, 11). Since only publicly available, published data were synthesized, research ethics approval and informed consent were not required.

We searched Ovid MEDLINE, Embase, and CINAHL from inception to June 30, 2025 using MeSH and non-MeSH terms (**Supplementary Document 1**). These databases were selected because these sources were expected to provide a high coverage of oncology, health services, and allied health literature, and report published evaluations of health-system and service-delivery interventions to improve EBRT access included in this review. The search strategy was developed in consultation with an information specialist. Reference lists of included articles were reviewed to ensure completeness, and forward citation searching was conducted to capture additional relevant publications. Covidence was used to conduct data screening and Zotero was used to manage referenced literature (12, 13).

We included English-language full articles and conference abstracts across all high-income and lower-middle-income geographic regions that assessed the implementation of a health-system or service-delivery interventions designed to improve access to EBRT for cancer patients. Eligible strategies encompassed infrastructure expansion such as the installation of new linear accelerators or development of treatment centers, workforce initiatives including training, recruitment, and task-shifting, telemedicine and digital innovations to support remote planning or referral, financing and policy reforms such as insurance coverage or public–private partnerships, patient navigation and outreach programs, and quality-improvement initiatives aimed at reducing treatment delays or enhancing machine uptime. Studies must have reported a primary outcome related to EBRT access to be included in this review. Acceptable study designs for inclusion included pre–post designs, interrupted time series, controlled cohort studies, cluster and non−randomized studies, and randomized trials. Studies that exclusively evaluated brachytherapy, translational research, or technical developments without a primary outcome related to access to EBRT were excluded. Reviews, commentaries, and study designs that did not report on a primary implementation of an intervention designed to improve access to EBRT in the study were excluded.

The primary outcome of interest for narrative synthesis was the equity of access to EBRT. We operationalized equity of access based on the Cochrane PROGRESS framework to map 6 domains of equity focus reported in at least one study, including socioeconomic status, rural-urban geographic location, sex, age, financial cost, and race or ethnicity (14, 15). These categories of equity focus were used to assign the domain of equity improvement intended to be addressed by the intervention. Secondary outcomes encompassed a defined set of system, financial, and patient-centered measures, including 1) capacity indicators such as the number of radiotherapy machines or treatment facilities per population, 2) workforce-related outcomes including training completion, staff retention, and task-shifting effects, 3) financial protection outcomes such as out-of-pocket spending, catastrophic health expenditure, or insurance coverage, and 4) patient-reported outcomes, such as satisfaction, treatment acceptability, and perceived quality of care. In addition, we captured operational and safety-related consequences when reported, including equipment downtime, machine maintenance, treatment interruptions, and quality lapses, as well as broader health system effects such as referral efficiency and integration with other oncology services.

Two reviewers independently screened titles and abstracts and full-text articles (K.X., R.L., K.A.). Disagreements were resolved through discussion with a third reviewer (D.C.). Data were extracted in duplicate using standardized forms. Extracted data included study characteristics (country, setting, design, sample size), intervention components, comparator group, outcomes measured, effect estimates, contextual details, and any unintended effects reported. We undertook a structured narrative synthesis among the screening team (K.X., R.L., K.A., D.C.), with at least 2 years of research experience and two certified radiation oncologists (F.Y.M,S.R.) with at least 7 years of practice experience, providing clinical input. We categorized interventions by type (infrastructure, workforce, financing, telemedicine, quality improvement) and summarized themes across primary and secondary outcomes (1, 16, 17). We also incorporated limited inductive refinement during data charting and synthesis when intervention components did not fit in the pre-specified domains within the *a priori* framework or spanned multiple domains. In such cases, studies were assigned a primary intervention category based on the dominant component of the intervention with reported outcomes.

## Results

**Figure 1:**
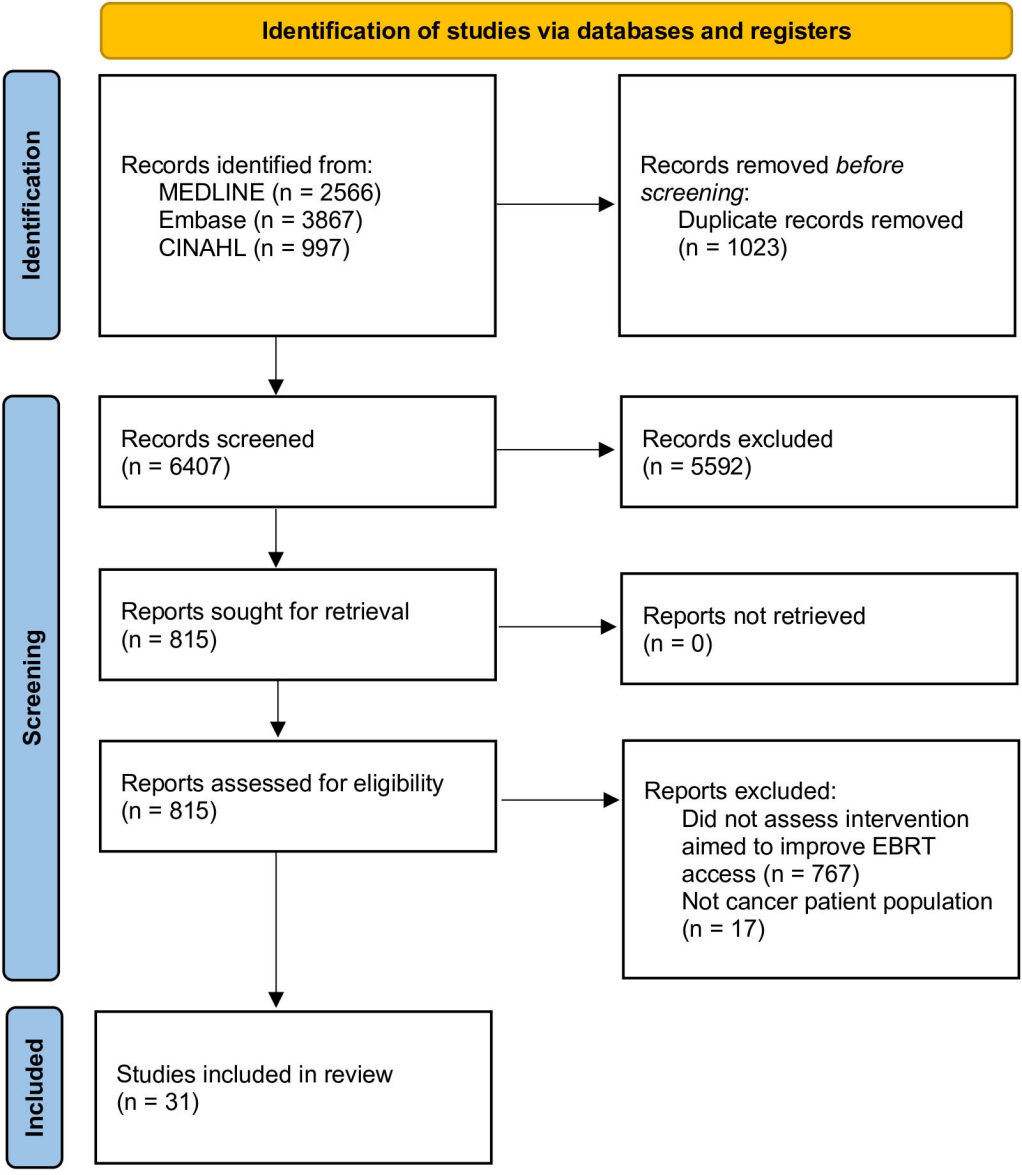
PRISMA diagram of inclusion and exclusion of studies.

The bibliographic search identified 7,430 records (**Figure 1**). Following the removal of 1,023 duplicates, 6,407 unique records were screened. After title and abstract review, 815 records were retained for full-text assessment. Of these, 784 were excluded, most commonly because they did not describe interventions aimed at improving access to EBRT. Ultimately, 31 studies comprised of 20 full articles and 11 conference abstracts met the inclusion criteria and were included in the synthesis (**Table 1**) (18–47).

**Table 1:** Primary characteristics of included studies.

Study ID	Country	WHO Region	Urbanicity	Setting	Equity Focus	Intervention Class	Intervention	Control	Access Outcome	Conclusion
Ashworth 2014 (19)	United States	AMRO	Urban	Institutional	Socio Economic Status	Financing	Free metro-cards provided to cover entire course of commuting for outpatient radiotherapy.	No Control	102/103 (99%) patients with free metro-cards completed planned RT. Overall compliance with scheduled sessions was 70%. 11/103 (10.7%) patients missed ≥1 session.	Eliminating transportation costs markedly improves radiotherapy compliance among underserved urban Medicaid patients.
Balbach 2024 (20)	Multi-National	AMRO + AFRO	Not Specified	International	Socio Economic Status	Workforce Capacity Building	27-session distance-learning IMRT curriculum (live Zoom + asynchronous materials) delivered free to 25 LMIC radiotherapy centres in Africa & Latin America.	No Control	Not Specified	Tele-education hub-and-spoke model feasible across languages. High live attendance (≥18 sessions) correlated with greater improvement in competency confidence. Language and technology access are barriers to distance learning.
Banjade 2020 (21)	Australia	WPRO	Rural	Institutional	Rural-Urban	Infrastructure	Commissioning and rollout of IMRT, VMAT and SABR (with 4D-CT, QA, staff training) at a rural cancer centre via quality improvement projects	Historical practice using 3DCRT before 2018.	Radiotherapy courses using IMRT/VMAT rose from 18% (2017, n=baseline) to 41% (2018) to 63.5% (2019) and 71.9% (Jan-2020). SABR comprised 2.1% (2018) and 2.2% (2019) of IMRT/VMAT cases.	Deployment of advanced technology is feasible in rural centres without extra staffing. Collaborative multidisciplinary leadership is essential.
Blain 2015 (22)	Canada	AMRO	Not Specified	Regional	Financial Cost	Service Delivery Innovation, Referral and Navigation	Telemedicine-based virtual radiation oncologist consultation for SRT via Ontario Telemedicine Network.	No Control	14/14 referred patients received virtual SRT consult. On average, each patient avoided 600 km travel and saved 7 hours journey time.	Booking coordination of telemedicine rooms remains main challenge. Dedicated telemedicine suite recommended for sustainability. Extensive planning and site readiness is essential.
Bosco 2023 (23)	Uganda	AFRO	Mixed	National	Rural-Urban	Infrastructure, Referral and Navigation	Scale-up of radiotherapy capacity (3 new external beam treatment machines, 9 radiation therapists) and Road to Care hostels and referral support for rural cervical cancer patients.	No Control	Radiotherapy throughput increased from 30 to 150 patients per month after scale-up. Road to Care pathway now facilitates treatment for 102 rural patients annually.	Local investment and non-government organization partnerships can rapidly restore and expand national chemoradiation capacity. Sourcing accessible accommodation is a priority for rural patients traveling away from home to receive radiotherapy treatment.
Burger 2022 (24)	South Africa	AFRO	Urban	Institutional	Socio Economic Status	Workforce Capacity Building	Hybrid 17-day Cape Town ‘Access-to-Care’ course: pre-course e-learning, VERT simulation, hands-on Eclipse planning, protocol development, 3-month remote mentorship to help African teams transition from 2D to 3DCRT.	No Control	Not Specified	Long delays between training and linac installation harm knowledge retention. Tracking progress increases online module completion. Multidisciplinary teamwork is required to address hierarchical barriers in facilitating improved collaboration between radiotherapy clinicians.
Butler 2017 (25)	Australia	WPRO	Rural	Regional	Rural-Urban	Infrastructure	Opening of first local radiotherapy centre in Orange, Western NSW Local Health District, replacing the prior distant referral model.	Historical control year 2010 before service opening when patients travelled to metropolitan centres/outreach clinics.	RTU rate: 2010 29.3% (573 courses) vs 2012 33.4% (667 courses), +4.1% (P=0.01); Mean travel distance: 338.7 km vs 210.2 km, −128.5 km (P<0.0001); Orange region RTU 30% vs 40%, +10% (P=0.0007); Remote region RTU 31% vs 20%, −9% (P=0.09).	Local radiotherapy service improves utilisation and reduces travel for nearby residents. Remote areas remain underserved, suggesting the need for additional complementary strategies.
Caicedo-Martinez 2023 (26)	Colombia	AMRO	Mixed	National	Socio Economic Status	Workforce Capacity Building	13-week country-wide e-learning curriculum on hypofractionation for radiation oncologists and physicists.	No Control	Paired radiation oncologists (n=38) selection of hypofractionation increased across all disease sites (mean score 6.2/12 to 8.2/12, P <.001). Paired clinicians (n = 87) self confidence increased for prostate ultrahypofractionation (+0.45), rectal ultrahypofractionation (+0.43), breast hypofractionation (+0.38), and prostate hypofractionation (+0.23) ( P ≤ .03).	Targeted, translated online curriculum increases clinician confidence and hypofractionation adoption. Scalable e-learning can improve radiotherapy access in LMICs.
Chapman 2007 (18)	Australia	WPRO	Mixed	Regional	Rural-Urban	Infrastructure	Establishment of single-machine radiotherapy units in three regional towns using oversight by metropolitan centres.	Historical pre-intervention period when patients travelled to metropolitan centres	Overall regional Victoria radiotherapy courses increased by 7.5% from 2002 to 2003, 63% in 2003 to 2004. Regional Ballarat radiotherapy courses increased from 0% in 2002 to 71% in 2003. Regional Bendigo radiotherapy courses increased from 0% in 2002 to 57% in 2003.	Hub-and-spoke single machine units can safely decentralise radiotherapy. Modern equipment and clear protocols improved documentation. Expansion of radiotherapy services should consider allied health needs and workforce expansion.
Chen 2023a (27)	United States	AMRO	Urban	Institutional	Rural-Urban, Socio Economic Status	Service Delivery Innovation	Free hospital-provided rideshare transportation to and from radiotherapy appointments.	Matched cohort of patients who did not use rideshare service.	More rideshare utilizers underwent RT for curative intent (79 vs 50%, p < .0001), received a higher number of fractions prescribed (median 28 vs 5, p < .0001), and completed their prescribed RT course (96 vs 81%, p = .01).	Utilization of free hospital-provided rideshare service was associated with improved RT completion rates.
Chen 2023b (28)	United States	AMRO	Not Specified	Institutional	Socio Economic Status	Financing	Institution implemented a pilot same-day access scheduling program offering new referrals consultation slots the day of initial contact.	Historical pre-initiative standard scheduling (no same-day offer).	Proportion of patients seen within 5 days: Blacks increased from 8% to 34%, Latinos 12% to 57%, Asians 18% to 67%, Caucasians 39% to 55% (p<0.001). Mean referral-to-consult days: Blacks 15 to 7, Latinos 12 to 5, Asians 10 to 3, Caucasians 6 to 4 (p<0.001). No-show rate: Blacks 20% to 7%, Latinos 14% to 5% (p<0.001).	Same-day appointment scheduling is feasible reduced racial disparities in radiotherapy access. Personalized human outreach and convenience as part of patient-centred model are important factors that promote equity.
Court 2024 (29)	United States	AMRO	Mixed	International	Socio Economic Status	Infrastructure, Service Delivery Innovation	Radiation Planning Assistant (web-based AI tool) auto-generating contours and radiotherapy plans for cervical, breast, head/neck and whole-brain cases.	No Control	Not Specified	RPA outputs judged clinically acceptable but requires final clinical review. Automation may alleviate staffing shortages. Physician variability influences acceptability.
Cuaron 2024 (30)	United States	AMRO	Mixed	Institutional	Rural-Urban, Socio Economic Status	Infrastructure, Financing, Service Efficiency	Fully remote physician management for radiotherapy via telehealth consults, remote planning, weekly virtual visits, treatment at nearest regional center.	No Control	2817 patients treated remotely Oct 2020–Oct 2022, comprising 10–15% of departmental volume. Each regional site had 10–35% of patients under remote model monthly.	Remote management is feasible and safe, preserves access, reduces financial toxicity and carbon footprint.
Erraisse 2019 (31)	Not Specified	EURO	Mixed	Institutional	Rural-Urban	Infrastructure, Workforce Capacity Building, Service Efficiency	Extended linear-accelerator operation to 24/7 including weekends, streamlined workflow, adopted hypofractionation to increase patient throughput.	Historical schedule 8 AM–7 PM weekdays only and conventional fractionation.	Waiting time from first consult to first fraction reduced from >3 months to ~2 weeks. Daily treatment slots increased from 50-60 to up to 100 patients.	24/7 operations, weekend treatments and hypofractionation can markedly shorten radiotherapy wait-lists when machine numbers are limited but requires adequate staffing.
Esho 2020 (32)	South Africa	AFRO	Not Specified	Institutional	Socio Economic Status	Infrastructure, Service Efficiency, Workforce Capacity Building	Radiation Planning Assistant autogenerated PMRT plans plus TPS-specific optimization guidelines to speed breast post-mastectomy planning.	Conventional fully manual contouring and planning.	Average uninterrupted planning time: Manual 120±60 min vs Pinnacle 13±11 min vs Eclipse 12±7 min, saving on average 108±51 min per plan.	Autoplanning plus concise guidelines yields consistent, clinically acceptable PMRT plans quickly in underserved clinics.
Graboyes 2021 (33)	United States	AMRO	Urban	Institutional	Rural-Urban, Race or Ethnicity	Referral and Navigation, Service Efficiency	NDURE: navigation-based multilevel program with patient education, travel support, standardized expectation discussions, post-operative radiotherapy care plans, referral tracking, and organizational role clarification.	No Control	86% of patients initiated radiotherapy in ≤6 weeks. Median surgery to post operative radiotherapy was 38 days (SD 7.7).	Navigation-based multilevel intervention feasible, acceptable, and may improve timely guideline-adherent post-operative radiotherapy and reduce racial disparities.
Jacobson 2018 (34)	Paraguay	AMRO	Mixed	Institutional	Rural-Urban, Socio Economic Status	Infrastructure, Workforce Capacity Building, Service Efficiency	Multifaceted QI: weekly multidisciplinary cervical cancer clinic, added pathologists, CT-based 3-D planning, HDR brachytherapy purchase, agreement with private linear accelerator	No Control	Median wait to start radiation reduced from 2–3 months to 4–6 weeks (~60% reduction). Pathology report turnaround reduced from 4–8 weeks to 1 week. HDR brachytherapy throughput increased from 2 to 16–20 cases/week.	Leadership-driven local solutions and multidisciplinary coordination can markedly improve radiotherapy access without large capital investment. Partnerships with NGOs, government and private sector accelerate capacity gains.
Joseph 2024 (35)	Multi-National	AFRO	Not Specified	International	Rural-Urban, Socio Economic Status	Workforce Capacity Building	Two-day (12 h) virtual Pediatric Radiation Oncology Course (PedROC) delivered via web-conferencing to African radiation oncology professionals.	No Control	African radiation oncology clinicians accessed pediatric radiation oncology education using virtual course approach.	Remote, technology-based training feasible and boosts pediatric RT capacity. Virtual format overcomes travel, cost, geographic barriers. Specialty-specific curricula recommended for sustained impact.
Kavuma 2023 (36)	Multi-National	AMRO + AFRO + WPRO	Mixed	International	Rural-Urban, Socio Economic Status	Workforce Capacity Building	36-day remote program combining synchronous lectures, synchronous hands-on case sessions, and asynchronous self-guided videos to train IMRT contouring, planning and QA for 37 radiation professionals in Uganda, Guatemala, Mongolia.	No Control	Uganda & Mongolia: IMRT clinical treatments commenced within 1 month post-training (baseline 0). Guatemala: daily IMRT cases increased from 30-40 to 90 patients/day after training.	Hands-on virtual sessions most preferred (59%). Complementary asynchronous+ synchronous model feasible across trainees worldwide. Language support and time-zone coordination critical for success.
Krishnatry 2024 (37)	India	SEARO	Urban	Institutional	Timely Access, Other	Service Efficiency	Quality-improvement workflow to ensure completed paperwork, machine availability confirmation the day before; treatment, and patient checklist for clear workflow instructions.	Pre-intervention baseline workflow (no advance paperwork).	Compared to baseline, median day 1 wait time reduced from 6 to 4.2 hours and the proportion of patients postponing treatment start to the second day of check in reduced from 22.5% to 2%.	Advance administrative completion and standardized communication significantly shorten wait. Multidisciplinary QI approach and leadership support critical for sustainable improvement.
Kyle 2019 (38)	United Kingdom	EURO	Not Specified	Institutional	Rural-Urban, Other	Referral and Navigation, Service Efficiency	Remote access to radiotherapy planning system provided to acute oncology team in cancer assessment unit.	Pre-intervention process using paper notes or leaving unit to access system.	Median request-to-decision time reduced from 25 min to 5 min post-intervention.	Providing remote electronic access to treatment information can markedly shorten decision times and improve staff satisfaction.
Lewis 2020 (39)	Multi-National	AFRO	Urban	Institutional	Socio Economic Status	Workforce Capacity Building, Service Efficiency	Cloud-based software platform enabling remote radiotherapy peer review and training across 4 African radiotherapy treatment centers.	No Control	Across 4 centers, software was installed successfully in 3/4 (75%) and complete feedback peer-review loop achieved in 1/4 (25%).	Cloud peer review feasible where internet adequate but needs improved bandwidth, staff training, and protected time before routine adoption.
Manning 2019 (40)	United States	AMRO	Not Specified	Institutional	Race or Ethnicity	Service Delivery Innovation	System-based package including real-time EHR alerts, race-stratified feedback, and nurse navigation to promote curative surgery/stereotactic radiation.	Retrospective and concurrent usual-care patients with no system intervention.	Baseline surgery/stereotactic RT treatment rates were 78% for Whites vs 69% for Blacks. Intervention cohort. Intervention curative surgery/stereotactic RT treatment rates were 95% for Whites vs 96.5% for Blacks.	Multi-component system intervention across five centers eliminated racial radiotherapy treatment gap.
McLaughlin 2016 (41)	Canada	AMRO	Mixed	Regional	Other	Referral and Navigation	On-site outreach clinics where radiation oncologists provide in-person consultations at hospitals lacking RT facilities.	Hospitals without RT facilities that offer no on-site radiation oncology consultations.	Hospitals with RO outreach had 29.8% patients receive RT within 1 year compared to 28.4% of patients of hospitals without outreach, RR 1.05 (95% CI 1.01-1.10. Hospitals with on-site RT had 34.1% patients receive RT within 1 year compared to 28.4% of patients of hospitals without on-site RT or outreach, RR 1.26 (95% CI 1.22-1.31).	On-site consultation availability yields small but significant increases in RT utilization. Provincial radiation oncology outreach activities vary widely.
Meshman 2020 (42)	United States	AMRO	Urban	Institutional	Other	Service Delivery Innovation, Service Efficiency	Electronic health record scheduling system including electronic follow-up orders, reserved staff time to reschedule no-shows, and verify appointments.	No Control	Follow-up appointments scheduled in EHR increased from 2% baseline to 98% at 2 months (p<0.01). No-show appointments rescheduled increased from 0% to 64% at 2 months (p<0.01). Patient no-show rate declined from 19% to 15% at month 2 (p=0.3).	Electronic scheduling can markedly improve appointment scheduling efficiency and may enhance retention in underserved oncology patients.
Ng 2024	Philippines	WPRO	Urban	Institutional	Financial Cost, Socio Economic Status	Service Delivery Innovation, Workforce Capacity Building, Infrastructure	International collaboration with GenesisCare Solutions providing remote dosimetry support, electronic medical record workflow, data reporting checklists, staff training and IT infrastructure upgrades at Fairview Cancer Center to improve radiotherapy access and efficiency.	No Control	IMRT utilisation rate increased from 20% to 54% post-intervention at 12 months. Mean patient wait time from check-in to treatment start reduced from 27 min to 17 min ( p≤0.001).	Virtual collaboration and electronic workflows can rapidly improve efficiency in LMIC radiotherapy centres. Remote planning support and structured training increase advanced technique adoption and data capture reliability.
Pearce 2013 (43)	Canada	AMRO	Mixed	Institutional	Rural-Urban	Infrastructure, Service Delivery Innovation	Creation of single-machine remote radiation unit (ADCP) with radiation oncologist coverage via telemedicine from tertiary centre.	Treatment at tertiary centre (Health Sciences North) compared to historical data.	90% of first-time patient consults were seen within acceptable target timeframe. Palliative radiation courses increased by 24% post-intervention. 86% of treatment reviews were completed by telemedicine.	Remote telemedicine-supported radiotherapy unit can deliver quality care, shorten waits, boost palliative access, and save travel costs.
Scott 2024 (44)	Ghana	AFRO	Urban	Institutional	Socio Economic Status, Rural-Urban	Infrastructure, Workforce Capacity Building, Service Delivery Innovation	Implementation of CT-based staging integrated with 3-D/IMRT planning and delivery for cervical cancer, enabled by new CT-simulator, IMRT-capable LINAC, and comprehensive staff training with Canadian collaboration.	Conventional 2-D EBRT planned by simulator after ultrasound staging and no CT imaging.	Out of 215 referrals, CT was obtained for 141 (66%), IMRT delivered for 56 (26%); CT staging upstaged for 57/91 (63%), and changed management for 61/91 (67%). Curative-intent treatment completion for IMRT was 47/48 (98%).	CT staging and IMRT is feasible in low-resource setting with technology investment and international mentorship. Tolerance, treatment compliance, affordability remains major barrier.
Sharma 2016 (47)	Australia	WPRO	Mixed	Regional	Rural-Urban	Infrastructure	Opening of a second radiotherapy facility (Radiation Oncology Queensland Cairns) in July 2011.	Historical pre-opening period (single facility, 2010-2011).	Prostate and breast patients receiving RT increased from 396 to 556 (40%) over 3 years. Median road distance travelled reduced from 270.5 km to 48.6 km (-82%). Median travel time reduced from 201 min to 56 min (−72%).	Adding regional RT centres improves utilisation and reduces travel burden.
Vieira 2023 (45)	Brazil	AMRO	Urban	Institutional	Socio Economic Status	Referral and Navigation	Patient navigation by providing scheduling, transport support, documentation assistance, and weekly phone follow-up from consent until completion of radiotherapy.	Historical cohort without patient navigation.	Median biopsy-to-RT start reduced 108 from 74 days (p<0.001). Proportion of patients starting RT ≤60 days increased from 20.5% to 38.5% (p=0.026). Biopsy result-to-RT referral time decreased from 53 to 40.5 days (p=0.011). Referral-to-first RT consult reduced from 25 to 13 days (p<0.001). Referral-to-RT completion reduced from 98 to 78 days (p<0.003).	Patient navigation optimization is low-cost, feasible, and reduces radiotherapy delays in Brazil.
Waran 2022 (42)	Malaysia	WPRO	Urban	Institutional	Socio Economic Status, Financial Cost	Infrastructure	Clinician-led, public-private partnership (PPP) to create the Centre for Image Guidance and Minimally Invasive Therapy (CIGMIT) at the University of Malaya with neurosurgical imaging and radiotherapy technology.	Conventional government hospital funded neurosurgical imaging and radiotherapy technology.	Conventional radiotherapy cases increased from 136 to 330. Stereotactic and IMRT cases increased from 48 to 207. Diagnostic CT scans increased from 0 to 5,899. Diagnostic MRI increased from 0 to 2,219.	Small, clinician-driven private-public partnerships can finance cutting-edge radiotherapy in LMICs. Shared public-private scheduling optimises utilisation.

The set of included studies represented a diverse geographical distribution across all World Health Organization regions, with the majority of studies designed in the Americas (AMRO) (n=14, 45%), Africa (AFRO) (n=6, 19%), and (Western Pacific) WPRO (n=6, 19%) regions (**Table 2**). Among the countries where the intervention was deployed, the most frequently represented countries included the United States (n=8, 26%), multiple countries (n=4, 13%), Australia (n=4, 13%), and Canada (n=3, 10%). Most evaluations of interventions were conducted at the institutional level (n=20, 65%), with fewer studies adopting regional (n=5, 16%), national (n=4, 13%), and international scope (n=4, 13%). The evidence base included in this review was predominantly comprised of access interventions implemented in higher-income environments, which should be considered when interpreting the generalizability of findings to lower-resource settings.

**Table 2:** Summary statistics of characteristics of included studies.

Category	Value	Count	Proportion
Equity Focus	Socio Economic Status	17	54.84
	Rural-Urban	15	48.39
	Financial Cost	3	9.68
	Race or Ethnicity	2	6.45
	Other	5	16.13
Intervention Class	Infrastructure	14	45.16
	Workforce Capacity Building	11	35.48
	Service Efficiency	9	29.03
	Service Delivery Innovation	8	25.81
	Referral and Navigation	6	19.35
	Financing	3	9.68
WHO Geographic Region	AMRO	14	45.16
	AFRO	6	19.35
	WPRO	6	19.35
	EURO	2	6.45
	AMRO + AFRO	1	3.23
	AMRO + AFRO + WPRO	1	3.23
	SEARO	1	3.23
Country	United States	8	25.81
	Multi-National	4	12.9
	Australia	4	12.9
	Canada	3	9.68
	South Africa	2	6.45
	Paraguay	1	3.23
	Uganda	1	3.23
	Brazil	1	3.23
	Philippines	1	3.23
	Colombia	1	3.23
	Malaysia	1	3.23
	Ghana	1	3.23
	United Kingdom	1	3.23
	Not Specified	1	3.23
	India	1	3.23
Intervention Setting	Institutional	20	64.52
	Regional	5	16.13
	International	4	12.9
	National	2	6.45

**Figure 2:**
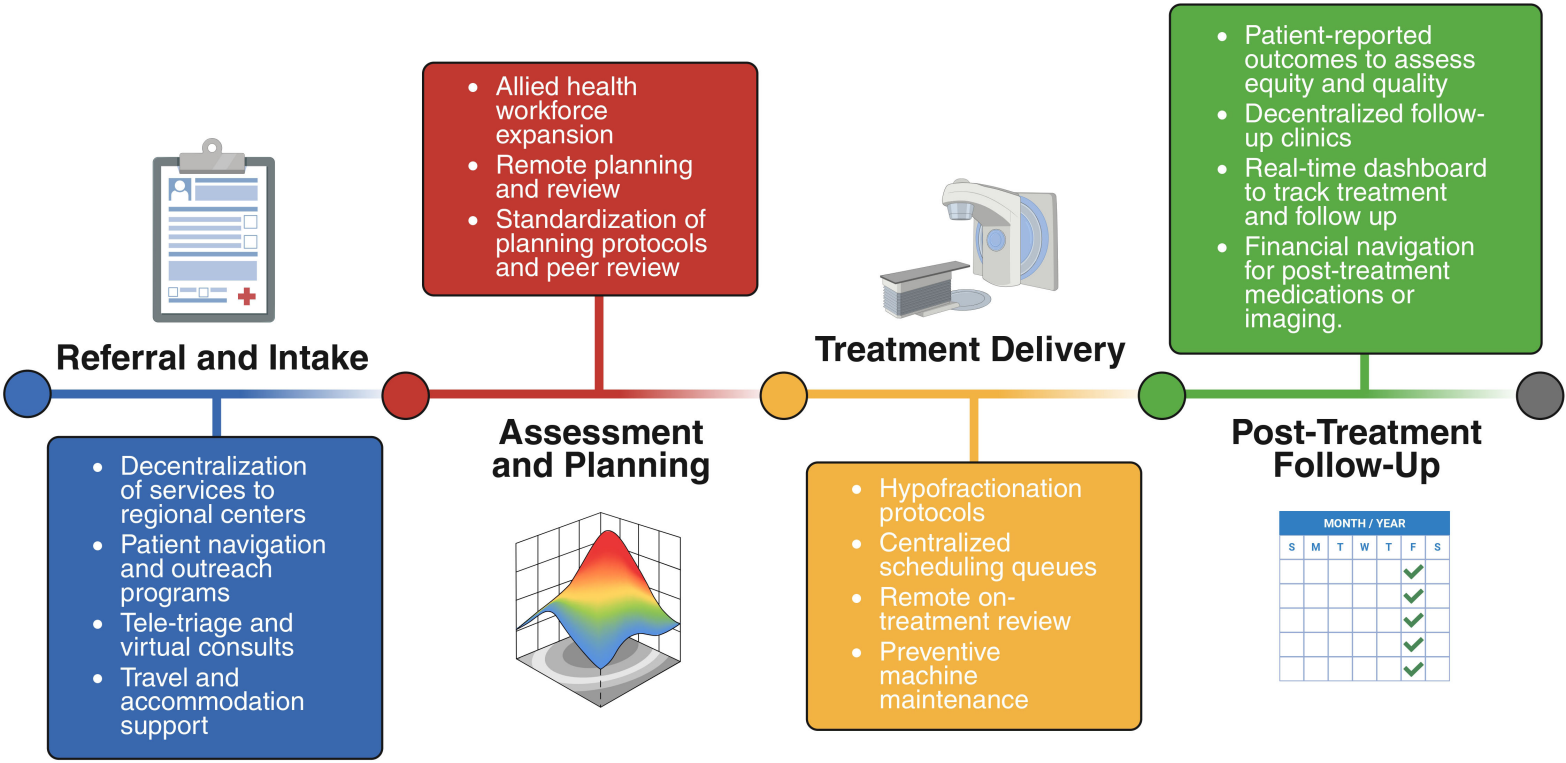
Summary of interventions designed to improve access to EBRT across the journey of a cancer patient receiving EBRT.

The interventions to improve EBRT access aimed to improve several dimensions of equity, such as socio-economic status (n=17, 55%), rurality (n=15, 48%), financial cost (n=3, 10%), and race and ethnicity (n=2, 6%). Inspired by the consensus of categories of interventions intended to improve access to radiotherapy interventions described in Atun et al., 2015 (1), Vandemaele et al., 2023 (17), and Laskar et al., 2022 (16), we grouped studies in our review into six major classes based on the primary intervention. These interventions involved infrastructure expansion (n=14, 45%), workforce development (n=11, 35%), service efficiency (n=9, 29%), service delivery innovations such as telemedicine (n=8, 26%), referral and navigation (n=6, 19%), and financial reforms (n=3, 10%) that span the journey of a cancer patient receiving radiotherapy treatment (**Figure 2**).

Infrastructure initiatives included the installation of new linear accelerators or the establishment of additional treatment facilities. Workforce development interventions focused on the training of radiation oncologists, physicists, and technologists, as well as task-shifting strategies. Service delivery reforms are frequently aimed at improving operational efficiency, for example, through quality improvement initiatives or redesigned scheduling systems. Telemedicine interventions primarily involved remote planning, contouring, and peer review. A smaller number of studies assessed financial interventions, such as programs to reduce out-of-pocket costs or establish sustainable public–private financing models.

The most common themes reported among the included studies are synthesized in **Table 3**. The majority of included studies reported improvements in access to EBRT, defined by increases in treatment utilization, reductions in waiting times, and higher rates of treatment initiation or completion. Several studies demonstrated improved equity in access, particularly for rural patients or those of lower socioeconomic status who otherwise face unique access challenges compared to higher-income, urban settings with greater access to radiotherapy resources. Interventions focused on operational redesign to improve workflow inefficiencies using the same set of radiation resources and personnel demonstrated the potential to reduce referral-to-treatment intervals and improve throughput of radiotherapy courses to treat a higher number of patients within a given time period.

**Table 3:** Common themes reported among included studies.

Theme	Description
Multi-component interventions	Integrated interventions that combine infrastructure, workforce training, and operational redesign demonstrate the most consistent improvements in EBRT access.
Decentralization and outreach	Establishment of regional or satellite centers and structured outreach increased utilization and improved access for rural populations.
Operational redesign	Service delivery innovations such as patient navigation, scheduling redesign, and quality improvement initiatives reduced treatment delays and improved timeliness.
Targeted financial supports	Interventions that reduce direct patient costs, such as transport vouchers or subsidized travel, increased treatment initiation and completion among socioeconomically disadvantaged patients.
Tele-radiotherapy	Telemedicine-enabled programs for remote planning, contouring, and peer review expanded specialist reach, although technical and governance barriers constrained scalability.
Hypofractionation and protocolization	Implementation of hypofractionation and standardized treatment pathways increased throughput and efficiency without compromising treatment quality or safety.
Measurement gaps	Despite frequent equity framing, outcomes related to equity, financial protection, and patient experience were under-measured, limiting the evidence base for policymaking.

Reporting of secondary outcomes was heterogeneous, as shown in **Supplementary Table 1**. Studies often described gains in technical capacity, such as the addition of linear accelerators or new simulation facilities, as well as improved workforce capacity outcomes, including increased training participation and retention of highly skilled personnel. Patient-reported and clinical outcomes were infrequently measured but were generally favorable, with no increase in reported toxicity when interventions such as hypofractionation or accelerated radiation treatment planning models were implemented. A minority of studies explicitly reported financial outcomes, cost considerations, or measures of economic sustainability, and very few documented unintended consequences, implementation barriers, or longer-term maintenance requirements.

## Discussion

This scoping review synthesizes available evidence on health system interventions designed to improve access to EBRT, which is a cornerstone of modern cancer care. The findings affirm that radiotherapy access can be meaningfully improved through deliberate, system-level interventions across infrastructure, workforce, financing, and service delivery domains. The breadth of included studies, ranging from high-income countries with underserved populations to LMICs with profound capacity shortages, underscores the universality of access challenges, while also highlighting the diversity of approaches pursued across the literature to address them.

We acknowledge that the included evidence in this review was disproportionately drawn from high-income countries, urban settings, and single institutions. This imbalance limits the generalizability of findings to low-resource (48, 49) and rural (50, 51) settings, where the constraints shaping access to EBRT are often related to limited capital investment, unreliable maintenance systems, workforce shortages, regulatory barriers, and weaker referral networks. As a result, interventions that appear feasible and effective in well-resourced settings, such as workflow redesign, same-day care pathways, or telemedicine-enabled subspecialty coordination, may require substantial adaptation before they can be implemented at scale in LMIC or rural contexts.

A key observation is that the majority of interventions reported access improvements, often measured as increased utilization, reduced waiting times, or improved completion of prescribed treatment. This finding is important given that delays or failure to access radiotherapy translate directly into preventable morbidity and mortality, particularly for cancers where radiotherapy plays a curative role (3, 52). However, despite frequent framing of health system interventions to improve equity, measurable outcomes related to equity, financial protection, and patient experience remained under-reported. The review also highlights that most studies were performed at a single institution and were short in duration, with heterogeneous outcome definitions and limited reporting of unintended, harmful consequences. Nevertheless, several recurring themes emerged that collectively provide a coherent framework for understanding how radiotherapy access can be expanded in practice. These themes, including multi-component interventions, decentralization and outreach, operational redesign, targeted financial supports, tele-radiotherapy, hypofractionation and protocolization, and persistent measurement gaps, can serve as guiding principles when designing future interventions to improve EBRT access.

### Multi-component interventions

The most durable gains in EBRT access outcomes were associated with interventions that combined multiple domains of action. Efficient delivery of radiotherapy often requires multi-disciplinary coordination of care, and a bottleneck at any step, ranging from intake, planning, contouring, physics checks, machine uptime, or patient transport and accommodations, can neutralize the benefits of investment in another step. Thus, we hypothesize that EBRT programs that paired capital expansion with workforce development, referral pathway redesign, and quality oversight may be better positioned to convert potential capacity into real-world improvements in treatment access and completion rates. To this point, Bosco et al., 2023 reported that simultaneous investment in three new EBRT machines, expansion of the radiation therapist workforce, and strengthened referral pathways increased national throughput from 30 to 150 patients per day (23). Similarly, Banjade et al., 2020 combined the commissioning of advanced technologies with the adoption of hypofractionation, thereby increasing the number of courses delivered while also reducing treatment completion time (21). Although infrastructure expansion, when pursued in isolation, may be undermined by workforce shortages or inefficient workflows, these studies illustrate that comprehensive approaches across multiple domains align resources to maximize impact.

These observations resonate with longstanding evidence that health technologies achieve their intended benefits only when embedded within adequately staffed, well-governed delivery platforms (1). For policymakers, the implication is that radiotherapy scale-up must be planned holistically in a way that integrates physical capacity, human capital, and operational redesign. First, multi-component programs should consider the total cost of program ownership, given that predictable budgets for service contracts, parts, and power are as important as the initial capital purchase and should be accounted for to limit foreseeable machine downtime. Second, training to define role clarification between radiation oncologists, physicists, and therapists among allied radiation health team members can help teams adopt new workflows and maintain performance. Third, data-driven feedback should track both system-level health outcomes and equity-sensitive measures so that gains are not realized for select demographics only, such as urban or insured patients (53).

### Decentralization and outreach

Geography remains one of the most challenging barriers to EBRT access. Patients living outside major urban centers often face prohibitive travel distances, leading to delayed or incomplete treatment. Several included studies demonstrated that decentralization of services or structured outreach from tertiary centers increased utilization and completion of radiotherapy courses. Bosco et al., 2023 provided national evidence of this effect through expansion of regional capacity in Uganda (23), while Blain et al., 2015 illustrated that outreach through virtual stereotactic consults enabled rural patients to access specialized treatment without referral to metropolitan centers in Canada (22). These findings highlight the importance of redistributing services rather than concentrating new investments solely in major cities. Without such redistribution, infrastructure expansion risks reinforcing existing inequities by disproportionately benefiting urban populations. For LMICs, where most radiotherapy facilities remain clustered in urban centers, decentralization and outreach will be critical to ensuring that expanded capacity translates into population-level gains. When first consulted, simulation, nursing education, and elements of follow-up can occur closer to home, and when accommodation is needed for shorter periods due to consolidated visits, the probability of completing a full EBRT course may increase.

To operationalize the concept of decentralization through hub and spoke radiotherapy program models, tertiary hubs can provide standardized protocols, remote planning support, and periodic on-site mentorship to satellites. Satellites should commit to standardized data reporting and active participation in peer review to maintain quality consistency across the network (17). To complement the collaboration between radiotherapy professionals in the hub and spoke model, program leads should consider travel patterns and social support to facilitate practical patient needs that require attendance at central hubs. Consideration of patient accommodation, transport, and appointment scheduling as part of the overall radiotherapy treatment journey can yield meaningful differences in access outcomes related to timeliness and completion stratified by rurality and income (16).

### Operational redesign

Operational design can impact access to radiotherapy even in settings where sufficient machines and staff are available. Multiple studies in this review demonstrated that interventions such as redesigning referral pathways, instituting same-day consults and simulation, introducing navigation services, and standardizing handoffs reduced the time to treatment and increased the proportion of patients who completed planned courses. Ashworth et al., 2014 found that eliminating transportation barriers enabled nearly universal completion of prescribed courses in the USA (19). Blain et al., 2015 reported that telemedicine coordination reduced referral bottlenecks for stereotactic radiotherapy in Canada, facilitating timely coordination of care between the first-time consult to the initiation of radiotherapy (22). Other studies employing navigation programs, same-day clinics, and scheduling redesign consistently demonstrated reductions in referral-to-treatment intervals. Operational redesign can be relatively inexpensive compared to financing new radiotherapy machines and can often be implemented quickly, offering high value in resource-constrained environments facing specific bottlenecks that limit efficiency.

Revision of operational efficiency through iterative quality improvement initiatives should be considered a core element of scaling EBRT rather than an optional adjunct to infrastructure or workforce interventions. Measurement of EBRT operational efficiency requires the development of standardized, auditable metrics that can include time from referral to consult, consult to simulation, simulation to treatment start, treatment interruption days, and completion rates tracked and stratified by equity-based demographics. In resource-constrained environments, operational redesign can also compensate for structural limitations by improving productivity per existing asset through flexibility within the workforce that can better account for fluctuations in radiotherapy demand. In contexts where staffing is limited, such as LMICs and regional centers in high-income countries, cross-training represents a critical, evidence-supported strategy to build surge capacity. Cross-training radiation therapists or dosimetrists to perform basic contouring for standard disease sites, or physicists to conduct secondary plan checks for straightforward techniques, allows services to maintain continuity when key personnel are unavailable (54). When implemented within clearly defined scopes of practice, under supervision, and with formal competency assessment, operational redesign through task sharing enables EBRT programs to address fluctuations in demand or temporary staffing gaps without compromising safety or quality.

Moreover, financial barriers are a decisive determinant of whether nominal access to EBRT is realized as real-world access to patients. Our findings are consistent with global surveys that position financial protection as a core pillar of equitable cancer control and universal health coverage, given that the financial burden of cancer care can result in catastrophic expenditure. Interventions that reduce out-of-pocket costs of transport, accommodation, caregiving, and foregone wages are likely to have disproportionately positive effects to address the inequity of EBRT access. Future studies of access to EBRT should consider financial protection as a primary endpoint using standardized definitions of out-of-pocket spending and catastrophic health expenditure. Evaluations of financial interventions to improve EBRT access can assess implementation metrics that determine real-world effectiveness, including timeliness of disbursement, ease of enrollment, integration with navigation and social work services, and mechanisms for low-friction reimbursement or subsidization of costs. It must be noted that financial support to patients can lead to increased demand and usage of radiotherapy services, which then warrants supply-side investments to support the workforce and machine capacity so that increased affordability of radiotherapy translates into timely, high-quality treatment rather than longer queues (1).

A major gap in the included literature was the limited reporting of implementation barriers, unintended consequences, sustainability, and economic considerations relevant to real-world adoption. Primary implementation studies suggest that successful radiotherapy access interventions often depend on enabling conditions beyond the intervention itself, including formal peer review, digital infrastructure, interdisciplinary training, and ongoing mentorship (20, 39, 44). Sustainable scale-up requires broader institutional readiness involving commitment, cooperation, capacity, and catalyst to establish safe and reliable radiotherapy services (55). Economic and practical trade-offs were also rarely assessed. Recent studies such as Cuaron et al. showed that fully remote radiation oncology management could be delivered with high patient satisfaction, low harmful rates of adverse events, and meaningful out-of-pocket savings, while longitudinal cost analyses from the PARCER program demonstrate that implementation decisions should consider downstream toxicity-management and wage-loss costs rather than upfront treatment costs alone (30, 56). Future evaluations of EBRT access interventions should therefore prospectively measure implementation barriers, safety, sustainability, and economic impact alongside access outcomes.

### Online radiation education and care

Beyond expanding the reach of existing EBRT programs, telemedicine and tele−education can change how expertise is distributed and supervised across national and international networks. Promising programs pair remote contouring, plan review, and virtual tumor boards with structured curricula and competency tracking so that the transfer of skills between personnel is measurable and durable. Balbach et al., 2024 implemented a multi-national distance-learning program that engaged 332 staff across 25 centers, creating IMRT capacity in both Africa and Latin America (20). There exists great potential for telemedicine to amplify specialist capacity and extend complex treatment planning into previously underserved regions. However, several programs cited connectivity limitations, insufficient governance, and variable regulatory frameworks as constraints. Successful initiatives combined digital innovations with workforce training, quality assurance, and structured mentoring, reinforcing that telemedicine is associated with improved access when integrated into broader systems of care rather than pursued as a stand-alone solution. This finding in our review is consistent with international calls for digital oncology infrastructure that prioritizes interoperability, security, and mentorship (57).

Recent international experience reinforces that the success of telemedicine in radiotherapy depends not only on technological readiness but also on institutional culture and sustained human mentorship. The International Atomic Energy Agency’s (IAEA) *Tele-Radiotherapy Network* demonstrated that remote contouring, plan peer review, and second-opinion networks can strengthen clinical decision-making, reduce planning errors, and accelerate the adoption of advanced techniques when embedded in national cancer control strategies (6, 58). In LMIC settings, tele-mentorship programs can connect isolated practitioners to subspecialist expertise and reduce workforce shortage without the need to establish local educational training with high capital costs. In high-income settings, by contrast, the focus has shifted toward hybrid tele-oncology that leverages remote planning and follow-up to enhance efficiency and patient convenience without displacing in-person, multidisciplinary collaboration. Across different income settings, the unifying lesson is that telemedicine should be viewed as the connection that links centers of radiotherapy excellence with emerging local programs, advancing both quality and equity in global radiotherapy delivery.

### Hypofractionation and standardization of protocols

Adoption of evidence−based hypofractionated regimens and standardized protocols was associated with increased radiotherapy throughput in several implementation reports. While randomized trials in breast (59), prostate (60), and lung (61) cancers have established that selected hypofractionated schedules yield comparable clinical outcomes to conventional fractionation, the implementation studies in this review primarily reported access−related metrics and rarely measured clinical endpoints necessary to suggest non-inferiority in the reported settings. Accordingly, we interpret hypofractionation’s contribution to access to RT as one dimension of its utility contingent on appropriate technology, training, and quality assurance.

Safe implementation of hypofractionation depends on having the appropriate technology and expertise to replicate the treatment protocols delivered in the clinical trials that have generated the evidence for hypofractionation. In resource-constrained settings, adopting simplified, evidence-based regimens for common disease sites, supported by international mentorship and established society and guideline-concordant methodology, offers a feasible entry point into hypofractionated radiotherapy delivery (6). Programs that embed hypofractionation within structured quality assurance frameworks, including prospective plan review and toxicity monitoring, may improve rates of treatment interruptions and adverse events. Taken together, hypofractionation may be a scalable, non-inferior strategy to expand global EBRT access for select disease sites and align delivery of resource-intensive radiotherapy with the broader goals of equity, quality, and universal health coverage.

### Future directions

The evidence synthesized in this review points to several priorities for future research and implementation of health system interventions to improve EBRT access. First, future studies should prespecify standardized endpoints and routinely stratify outcomes by socioeconomic status, geography, gender, and other relevant equity-deserving axes. For example, the ESTRO value-based radiation oncology categorization system introduces a mixed-methods approach to defining radiotherapy interventions, which can be useful as a model towards future development of value-based frameworks for interventions to improve radiotherapy outcomes and standardized endpoints (62). Coupled with standardized endpoints, developing data-driven dashboards that track utilization, timeliness, and completion across population subgroups could generate high-quality evidence based on clinical and access outcomes (63).

Second, financial protection should be considered as an important consideration of intervention design and evaluation. Out-of-pocket expenditure and catastrophic health spending should be treated as core outcomes, especially in LMIC settings where financial burden disproportionately affects patient engagement. Sustainable financing models, including public–private partnerships, insurance reforms, and targeted subsidies, should be rigorously assessed to ensure both affordability for patients and fiscal sustainability for health systems (6).

Third, tele-radiotherapy and digital oncology innovations should be scaled in tandem with infrastructure and workforce investments. To succeed, such programs must be embedded in quality assurance frameworks and supported by reliable connectivity, governance, and mentorship. International collaborations and training networks can play a pivotal role in ensuring that digital platforms are readily integrated into existing patient care workflows. Finally, hypofractionation and protocolized workflows should be prioritized as promising efficiency strategies with strong potential to expand capacity while considering risks of adverse outcomes and safety. Implementation should be accompanied by workforce training, peer review, and safety monitoring to ensure consistent quality.

Overall, adopting value-based radiation oncology strategies modeled after value frameworks used to appraise systemic cancer therapy, such as the American Society of Clinical Oncology Value Framework and Magnitude of Clinical Benefit Scale by the European Society for Medical Oncology, serves as a promising direction towards designing access interventions that improve health outcomes that matter most to cancer patients receiving radiotherapy (64–66). Radiotherapy interventions are advancing rapidly with the commendable aim to improve patient outcomes. However, adopting these innovations too hastily without real-world, value-based evidence risks overlooking the fundamental issues related to radiotherapy underutilization and access. Inspired by the ESTRO-HERO initiative, we encourage future interventions that aim to improve access to EBRT to define the primary purpose and equity domain addressed by the intervention, define standardized outcomes that support its implementation, and pre-specify the magnitude of benefit and evidence required to appraise its feasibility and efficacy (67).

### Limitations

This review is subject to several limitations. We only included primary studies of interventions intended to improve access to EBRT and reported a primary outcome related to access to EBRT. Conference abstracts comprised 11 out of 31 of the included sources, potentially introducing selection bias and incomplete outcome reporting. The included studies were heterogeneous in design, often reported as pre–post studies without concurrent controls making it difficult to separate the effects of the intervention from background improvements in health services or random, short-term fluctuations in outcomes that naturally return toward baseline values over time. Moreover, heterogeneity in reported outcomes and context precluded quantitative meta-analysis and synthesis. Most included studies were institutional, often limiting their generalizability to national and international contexts. Outcome definitions were inconsistent, and secondary outcomes such as clinical outcomes, patient-reported outcomes, and financial metrics were under-reported. The geographic distribution of studies was skewed toward high-income countries, with fewer studies conducted in LMIC and rural settings that limits the generalizability of this review’s results. We acknowledge that publication bias toward successful pilot projects may highlight interventions that tended to succeed in improving access to EBRT.

Although equity and access were commonly identified as outcomes of health system-level interventions to improve EBRT access, the absence of standardized outcome definitions further constrained synthesis and comparability across studies. Access to EBRT was variably defined across studies, ranging from utilization counts to timeliness or adherence metrics. Clinical outcomes, adverse events, and unintended consequences were often rarely reported. These gaps likely reflect the pragmatic nature of service improvement evaluations, but they limit the generalizability of findings needed to compare studies across different contexts. To inform national scale-up efforts, future studies must move beyond descriptive pilot reports toward prospective, rigorously designed evaluations with prespecified access and equity endpoints.

## Conclusion

This scoping review synthesized evidence from 31 studies evaluating health system interventions aimed at improving access to EBRT. Across diverse contexts, interventions were most often associated with improvements in treatment utilization, timeliness, and completion. The most effective strategies include multi-component approaches that combine infrastructure expansion, workforce development, and operational redesign. Additional promising strategies included decentralization of services to address geographic inequities through hub-and-spoke models of care, targeted financial supports to reduce out-of-pocket costs, and tele-radiotherapy initiatives to extend specialist capacity and outreach. Hypofractionation and standardized protocols were shown to enhance throughput while maintaining non-inferior clinical outcomes without compromising safety. Collectively, the results of this review suggest that carefully designed, system-level interventions can meaningfully expand radiotherapy access, but future prospective, equity-focused evaluations are required to guide sustainable, collaborative radiotherapy scale-up in national and international contexts.

## Supplementary material

Supplementary Table 1 Secondary outcomes reported among included studies.
